# Chemical Markers for Differentiating Yellow Prickly Pear (*Opuntia ficus-indica*) from Southern Greece: Insights from Physicochemical Parameters, Elemental Composition, Antioxidants, and Vitamins

**DOI:** 10.3390/molecules30112448

**Published:** 2025-06-03

**Authors:** Artemis P. Louppis, Michael G. Kontominas, Michalis S. Constantinou, Ioanna S. Kosma, Anastasia V. Badeka, Georgios Stamatakos

**Affiliations:** Laboratory of Food Chemistry, Department of Chemistry, University of Ioannina, 45110 Ioannina, Greece

**Keywords:** prickly pear, geographical differentiation, physicochemical parameters, mineral composition, antioxidants, Vitamins, Greek

## Abstract

This study presents an innovative approach to differentiate Southern Greek yellow prickly pear samples according to geographical origin based on physicochemical parameters, mineral composition, and bioactive compounds using advanced chemometrics. A total of 56 yellow prickly pear samples were collected from four distinct Greek regions (Crete, Paros, Symi, Peloponnese) during the 2019 and 2020 harvest seasons. A multi-platform analytical strategy was employed, combining classical physicochemical analyses and UV spectrophotometry for total antioxidant activity with cutting-edge techniques such as UPLC-MS/MS for precise quantification of vitamins and antioxidants, and ICP-MS for mineral profiling. In total, thirteen physicochemical parameters, nineteen macro-, micro-, and trace elements, nine vitamins, and seven antioxidants were identified and quantified. Application of MANOVA and Linear discriminant analysis (LDA) revealed that eight physicochemical parameters, ten mineral elements, and sixteen bioactive compounds played a crucial role in sample geographical differentiation. The classification success rates using the cross-validation method were 82.1% for physicochemical parameters, 75.0% for minerals, and an impressive 96.4% for vitamins and antioxidants highlighting the robust tool for the geographical differentiation of Southern Greek yellow prickly pears.

## 1. Introduction

The genus *Opuntia Mill.*, commonly known as prickly pear cactus, includes various species that produce highly nutritious fruits and young, edible cladodes (stem pads). These plants are well-adapted to arid and semi-arid climates, exhibiting remarkable resilience to harsh environmental conditions. The most widely cultivated genera include *Opuntia* (subfamily *Opuntioideae*) and *Hylocereus* (*A. Berger*) *Britton & Rose* (subfamily *Cactoideae*) [1]. *Opuntia* belongs to the subfamily *Opuntioideae*, which encompasses several genera [2]. Among them, *Opuntia ficus-indica* (L.) *Mill.* is considered the most agro-economically significant cactus crop species [3,4,5]. In commercial markets, prickly pear fruits are available in both peeled and unpeeled forms and exhibit a variety of colors, including white, green, yellow (the most common), orange, red, and purple. These color variations are primarily attributed to differences in betalain pigment content [2,6]. Prickly pear cacti, native to the highlands of Mexico, have since spread to various regions worldwide, adapting to diverse climates and soil conditions [7]. The *Opuntia* species, primarily *Opuntia ficus-indica Mill*., was introduced to the Mediterranean basin around five centuries ago from their region of origin [8]. Since then, they have progressively invaded natural habitats, particularly in Spain, Italy, Greece, and North Africa [9], and have been cultivated for multiple purposes. In Mediterranean countries, *Opuntia ficus-indica* is grown for forage and fodder, as well as for the commercial fruit industry. Beyond its economic importance, the prickly pear cactus is increasingly recognized for its ecological benefits. It is considered a valuable crop for re-vegetation purposes, helping to mitigate wind and water erosion in degraded landscapes [10,11]. Cactus plants have diverse applications, serving as sources of both food and raw materials. Their fruits and cladodes can be consumed fresh or processed into various products, such as candied fruit (glacé fruit), juices, jams, and alcoholic beverages. Additionally, they are used to produce natural colorants, dietary supplements, pharmaceutical products, cosmetic ingredients, animal feed, and even construction materials [5,6,8]. These multifunctional attributes underscore the significant agricultural, environmental, and commercial potential of *Opuntia ficus-indica* worldwide.

The geographical origin of food has long played a significant role in determining its quality and authenticity. Historical records indicate that specific food products have been associated with particular regions, as seen in Ancient Egypt, as well as with iconic foods such as Parmigiano Reggiano in Italy, Stilton in the UK, Comté in France, and Washington apples in the USA [12]. Today, food quality and authentication have become crucial for both consumers and industries, encompassing all stages of the production process, from raw materials (farm) to final products (fork). Such authentic specialty products, i.e., fruits and vegetables enjoy higher prices both nationally and internationally compared to respective conventional products due to their perceived authenticity and superior quality. Given the wide range of factors that influence food authenticity, including natural cultivar variation, regional climatic differences, storage conditions, sample handling, and processing techniques, selecting the appropriate analytical method is essential in order to accurately differentiate plant-based products according to their geographical origin. Various analytical techniques have been used for food authentication, ranging from simple methods such as measuring soluble solids, color score, and suspended pulp content to more advanced approaches that involve profiling sugars, organic acids, flavonoids, mineral content, etc., using high-performance liquid chromatography (HPLC) and gas chromatography (GC) coupled with various detectors [13,14,15], capillary electrophoresis [16], spectroscopy [17], inductively coupled plasma mass spectrometry (ICP-MS) [18], neutron activation analysis [19], and isotope ratio mass spectrometry (IRMS) [20].

Several studies have demonstrated the efficacy of these methods in food authentication. Åkerström et al. (2010) [21] analyzed anthocyanidin concentrations in *Vaccinium myrtillus* (bilberries) using HPLC to evaluate the impact of geographical origin and genetic variation. The results showed that anthocyanidin concentrations in bilberries were influenced by both climatic conditions and genetic factors. Similarly, carotenoid levels in apricots, which are typically higher in Mediterranean-grown fruit, are strongly affected by both cultivar and region of cultivation. Li et al. (2024) [22] investigated the stable isotope ratios and elemental profiles of Jingbai pears, along with those of the corresponding soils and groundwater, from four regions in China. Using isotope ratio mass spectrometry (IRMS) and inductively coupled plasma mass spectrometry (ICP-MS), they identified significant regional differences in δ^15^N, δ^18^O, and lithium (Li), achieving a 92.3% of classification based on geographical origin. Metal content analysis has also been used as a means of geographical differentiation, as demonstrated by Lo Turco et al. [23] in their study on dried fig samples collected from three Mediterranean regions (Greece, Turkey, and Italy), achieving a classification accuracy of 82.85%. Furthermore, Matos-Reyes et al. (2013) [24] employed ICP-OES to analyze 28 Spanish fruit varieties, further emphasizing the role of mineral profiling in food authentication. Despite the extensive research on botanical and geographical differentiation of various fruits and vegetables, studies focusing on the geographical classification of prickly pear (*Opuntia ficus-indica*) remain limited [6,25,26,27,28]. While previous studies have explored the nutritional composition, bioactive compounds, and health benefits of prickly pear [11,29,30,31], little attention has been given to its chemical fingerprinting in relation to its geographical origin. Given the increasing economic importance of prickly pear in the Mediterranean region especially in Italy, Spain, and Greece, a reliable analytical method for its geographical differentiation is essential for quality control, consumer confidence, and protection against food fraud. This study addresses this gap by integrating analysis of physicochemical parameters, mineral profiling, and targeted bioactive compound analysis with chemometrics to establish a robust classification model for Southern Greek yellow prickly pears.

## 2. Results and Discussion

### 2.1. Conventional Physicochemical Parameters Including Color and Sugar Content of Yellow Prickly Pears

Data on the conventional quality parameters of yellow prickly pears are presented in Table 1. The analytical characteristics for sugars are shown in Supplementary Material Table S1. Significant differences (*p* < 0.05) were observed among the samples for pH, electrical conductivity (EC), moisture content, total carbohydrates, fructose, and glucose. The pH values ranged from 6.13 ± 0.19 in samples from Paros Island to 6.35 ± 0.14 in samples from the Peloponnese. EC values varied from 151 ± 26 μS/cm in Cretan prickly pears to 202 ± 21 μS/cm in samples from Paros Island. Moisture content ranged from 78 ± 4% in Cretan samples to 84 ± 2% in Symian samples. Total carbohydrates content differed significantly (*p* < 0.05) among samples, with values ranging from 11 ± 1% in Symian prickly pears to 18 ± 4% in Cretan samples. Similarly, fructose content varied from 1.25 ± 0.61% in Symian samples to 3.69 ± 1.17% in those from Paros Island, while glucose content ranged from 0.82 ± 0.06% in Symian samples to 4.36 ± 1.37% in Paros Island samples. In contrast, titratable acidity (TA), crude protein, ash (acid-insoluble), and crude fat on a dry weight basis did not show statistically significant differences (*p* > 0.05) among the four regions studied. Symian samples exhibited the highest TA (0.079 ± 0.02 g/100 g), while Peloponnesian samples recorded the highest crude protein content (1.15 ± 0.42%) and the highest crude fat content on a dry weight basis (2.51 ± 0.83 g/100 g dry weight basis). Ash content ranged between 0.55 ± 0.04 g/100 g recorded in samples from Symi Island and 0.62 ± 0.11 g/100 g recorded for Paros Island. Table 1 presents the results of the objective color measurement of Mediterranean prickly pear samples. According to the HunterLab System, color can be defined based on three color parameters: (L), (a), and (b). These parameters are based on the opponent color theory according to which the human eye perceives color as the result of light–dark or Lightness (L), red–green or redness (a) and yellow–blue or yellowness (b) combinations, where red hues correspond to (+a) and green hues to (−a), while yellow hues correspond to (+b), and blue ones to (−b). Regarding Lightness values (L), the samples from Crete, Symi appeared to be lighter (65.68 ± 1.21, 65.40 ± 3.46, respectively), while the samples from Peloponnese and Paros were somewhat duller (62.66 ± 4.20, 61.34 ± 2.59, respectively). In terms of redness (a), the samples from Symi, Peloponnese, and Crete showed more green shades (−3.21 ± 1.42, −1.79 ± 1.86, and −1.54 ± 1.27, respectively) than the samples from Paros (0.91 ± 3.32), while in terms of paleness, the samples from Symi appeared to be more yellow (1.04 ± 0.56).

Physicochemical parameters including moisture content (91.1%), protein (1.7%), fat (0.1%), ash (5.4%), and total carbohydrates (92.7%) in yellow prickly pears from Mexico were determined by Valero-Galván et al. (2021) [32]. The values reported for these parameters were higher than those determined in the present study. On the other hand, Cota-Sánchez (2016) [33] and Díaz-Medina et al. (2007) [34] analyzed the same physicochemical parameters in prickly pears. Cota-Sánchez (2016) [33] reported values 84–90% for moisture, 12–17% for carbohydrates, 0.3–1% for ash, and 0.21–1.6% for protein, while Díaz-Medina et al. (2007) [34] reported 82.61% for moisture, 0.94% for protein, and 0.37% for ash. The values reported for these parameters by Cota-Sánchez (2016) [33] and Díaz-Medina et al. (2007) [34] were very similar to those determined in the present study. Regarding pH and titratable acidity, Díaz-Medina et al. (2007) [34] reported values of 6.22 for pH and 0.086 g of anhydrous citric acid/100 g, which are consistent with the findings of this study (6.13–6.35, 0.064–0.079 g of anhydrous citric acid/100 g). Differences in physicochemical parameter values between the present study and those reported in the literature may be attributed to pedoclimatic variations, harvesting and maturity stages, as well as processing and sample preparation methods.

### 2.2. Minerals of Yellow Prickly Pear

A total of nineteen macro-, micro-, and mineral elements were identified and quantified in prickly pear samples. The analysis was conducted using in-house methodologies based on certified standards. Elements such as K, Ca, P, Mg, and Fe were measured in standard mode, while B, Si, Zn, Mn, Na, Sr, Al, Cu, Ni, Ba, Sn, Ti, Mo, and Co were determined using the KED (Kinetic Energy Discrimination) mode. No significant spectral interferences were observed, allowing for the quantification of these elements using their most abundant isotopes. Additionally, specific correction equations were applied to eliminate interferences for certain elements (Fe: −0.028226 + Cr^52^; Mo: −0.109613 + Ru^101^; Ba: −0.002838Ce^140^ − 0.000901Ca). Matrix-matched calibration curves were generated within the concentration range of 0.1–10 mg/L for all elements, except for Sn, Ti, Mo, and Co, for which a range of 0.01–1 mg/L was used. The calibration curves exhibited good linearity, with correlation coefficient (R^2^) values exceeding 0.99 (Supplementary Material, Table S3). Method accuracy was assessed through the analysis of quality control samples and a certified reference material (CRM) (FCCM23-FRU51RM). Recovery rates ranged between 85% and 98% for both quality control and CRM samples. Additionally, the limits of detection varied from 0.001 to 4 mg/kg (Supplementary Material, Table S2).

According to data shown in Table 2, the most abundant elements in yellow prickly pears were K, Ca, P, and Mg, while Cu, Al, Ni, Ba, Sn, Ti, Mo, Co, B, Si, Zn, Mn, Na, and Fe were present in lower concentrations. Notably, the abundance order of the major minerals (K > Ca > P > Mg) and the least abundant element (Co) remained consistent across all samples from the four regions studied. The highest concentration was observed for K, ranging from 1794 to 2237 mg/kg, whereas Co was the least abundant element, with concentrations ranging from 0.0016 to 0.0038 mg/kg. Overall, the total mineral content in yellow prickly pear samples followed the descending order: Peloponnese (5360 mg/kg) ≥ Symi (5309 mg /kg) > Paros (5272 mg/kg) > Crete (4610 mg/kg).

Nine minerals in American prickly pears were determined by Mayer et al. (2020) [35]. Among these, the concentrations of P (24 mg/kg), K (1457 mg/kg), Cu (0.055 mg/kg), Mn (1.11 mg/kg), and Zn (0.54 mg/kg) were lower than those found in the present study, while the concentrations of Ca (5705 mg/kg), Mg (1621 mg/kg), Na (12 mg/kg), and Fe (2.84 mg/kg) were higher. Karabagias et al. (2019) [25] analyzed the mineral content in Greek yellow prickly pear juice, reporting values for B (1869–2398 mg/kg), K (2240–2630 mg/kg), Al (0.26–0.45 mg/kg), and Mg (93.92–108.50 mg/kg). The concentrations of K and Al were similar to those found in the present study, whereas B and Mg were lower. Additionally, El-Sayed et al. (2014) [36] determined K levels (2088 mg/kg) in yellow prickly pears from Saudi Arabia, which were comparable to the concentrations (1794–2237 mg/kg) observed in the present study. Differences in mineral content between the present study and those reported in the literature may be attributed to variations in soil composition, climatic conditions, usage of fertilizers, harvesting stages, and analytical methodologies used.

### 2.3. Individual Vitamin and Antioxidant Content, TPC, TFC, and Antioxidant Activity of Yellow Prickly Pear

A targeted analysis was conducted to identify and quantify individual vitamins and antioxidants in yellow prickly pears. The determination was based on the availability of high-purity analytical standards, particularly for antioxidants. Nine vitamins (nicotinic acid, nicotinamide, pyridoxal, pyridoxine, and vitamins B1, B2, B5, B7, and C) and seven antioxidants (taurine, rutin, quercetin, catechin, isorhamnetin, gallic acid, and kaempferol) were determined, along with total flavonoids, total phenolics, and antioxidant activity (DPPH and FRAP assays) (Table 3). Analytical characteristics for the above parameters are shown in Supplementary Material Tables S3–S5. Among the vitamins, vitamin C was the most abundant, with concentrations ranging from 8,907 to 69,272 μg/kg, while pyridoxine was the least abundant, ranging from 2.12 to 6.38 μg/kg. Rutin was the most prevalent antioxidant (6–36 μg/kg), whereas gallic acid had the lowest concentration (0.49–1.39 μg/kg). Regional differences in vitamin and antioxidant content were observed. Peloponnesian yellow prickly pears had the highest concentrations of nicotinic acid, pyridoxine, vitamin B5, taurine, quercetin, isorhamnetin, gallic acid, and kaempferol. Symian samples recorded the highest levels of nicotinamide, pyridoxal, vitamins B7 and B2, while Cretan samples contained the highest concentrations of vitamin C, rutin, and catechin. Prickly pears from Paros Island had the highest vitamin B1 content. According to antioxidant assays, the highest antioxidant activity was recorded in prickly pears from Paros Island (FRAP = 62 ± 8 mg/kg FeSO_4_, DPPH = 16 ± 2 mg/kg Trolox), followed by Peloponnesian samples in the FRAP assay (50 ± 5 mg/kg FeSO_4_), and Cretan samples in the DPPH assay (9 ± 2 mg/kg Trolox). Additionally, samples from Paros Island recorded the highest total flavonoid content (6.20 ± 1.97 mg/kg quercetin), followed by Peloponnesian samples (4.72 ± 0.96 mg/kg quercetin). The highest total phenolic content (*p* < 0.05) was observed in Cretan samples (114 ± 19 mg/kg gallic acid), followed by samples from Paros Island (90 ± 12 mg/kg gallic acid). The total vitamin content followed the order Crete > Paros > Symi > Peloponnese, while antioxidant activity ranked as Crete > Paros > Peloponnese > Symi. Differences in antioxidant and vitamin values between the present study and those reported in the literature may be due to factors such as environmental conditions, agricultural practices, ripening stage at harvest, storage conditions, and differences in analytical methodologies used.

De Wit et al. (2019) [37] determined ascorbic acid (94.67 mg/100 g) and total phenolics (17.74 mg gallic acid/kg) in yellow prickly pears from South Africa. The ascorbic acid content was higher than that reported in the present study, while the total phenolics content was lower. In contrast, the total phenolics concentration (86 mg gallic acid/kg) reported by Amaya-Cruz et al. (2018) [38] in yellow prickly pears was comparable to the range observed in this study (70–114 mg gallic acid/kg). Finally, Albano et al. (2015) [39] determined vitamin C and total phenolics in Italian yellow prickly pears, reporting a vitamin C concentration of 30.2 mg/100 g and total phenolics of 70 mg gallic acid/100 g. Compared to the present study, both vitamin C and total phenolics concentrations were lower. These differences may be attributed to the same factors mentioned above.

## 3. Geographical Differentiation of Yellow Prickly Pears

### 3.1. Differentiation of Yellow Prickly Based on Physicochemical Parameters

**Figure 1:**
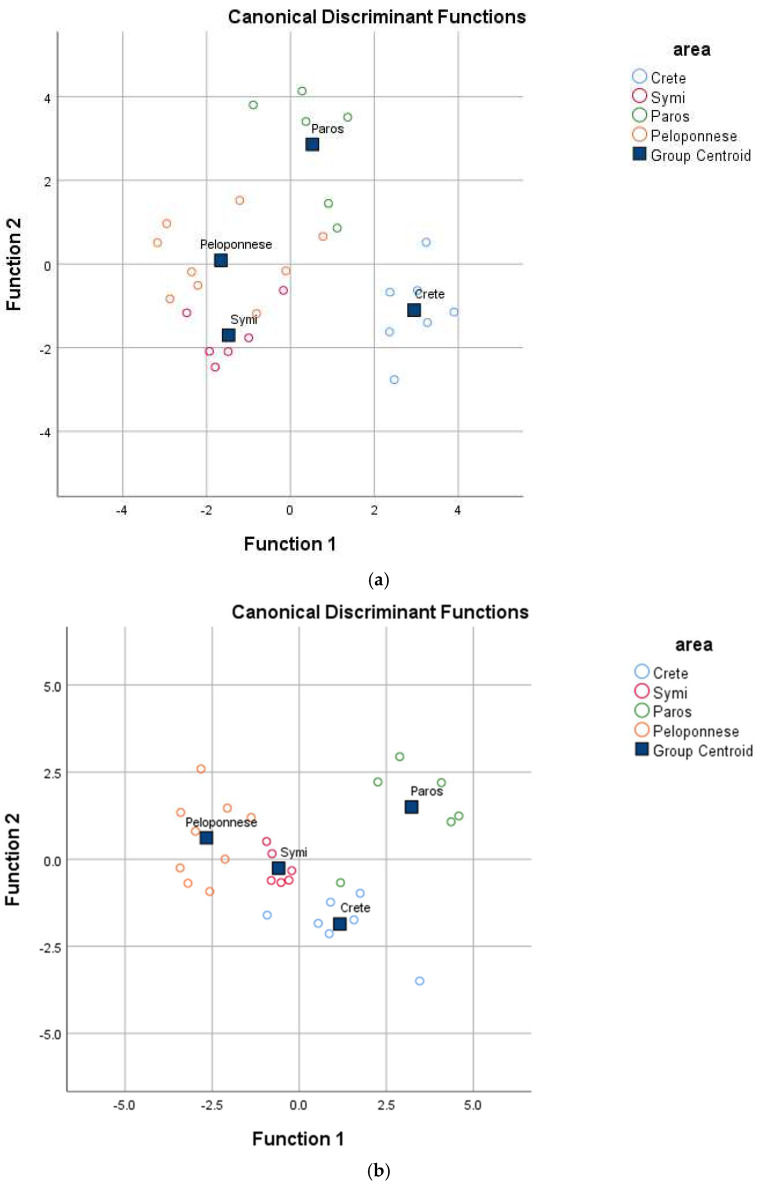

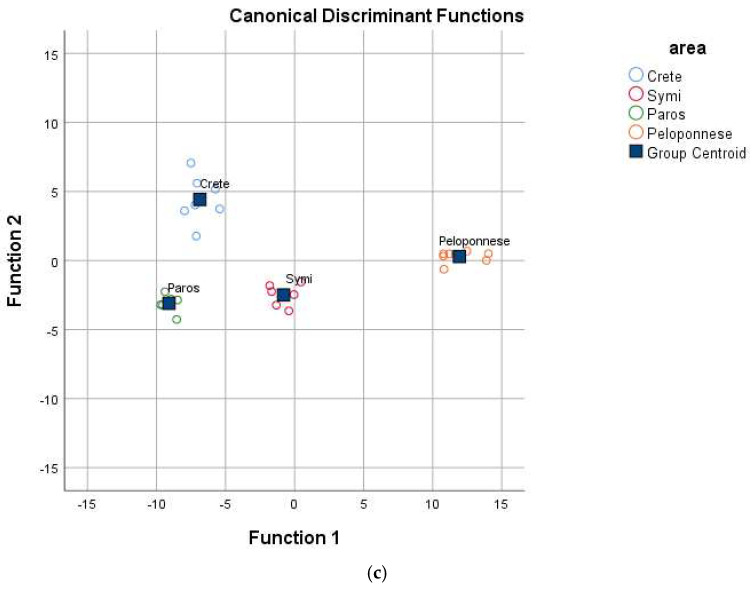
Geographical differentiation of Greek yellow prickly pear based on (**a**) physicochemical parameters, (**b**) mineral content, and (**c**) vitamin and antioxidant content.

The multivariable analysis confirmed that the geographical origin had a significant impact on the physicochemical parameters of prickly pear, as reflected by a Pillai’s Trace value of 2.128 (F = 2.629, *p* = 0.001 < 0.05) and a Wilk’s Lambda value of 0.014 (F = 3.012, *p* = 0.001 < 0.05). Eight analytical parameters significantly (*p* < 0.05) contributed to the differentiation of yellow prickly pears and were subsequently analyzed using Linear Discriminant Analysis (LDA) (Table 1). The results revealed the formation of three statistically significant discriminant functions (Wilk’s Lambda = 0.029, X^2^ = 74.272, df = 24, *p* = 0.000 < 0.05 for the first; Wilk’s Lambda = 0.151, X^2^ = 39.738, df = 14, *p* = 0.000 < 0.05 for the second; and Wilk’s Lambda = 0.622, X^2^ = 9.974, df = 6, *p* = 0.013 < 0.05 for the third). The test for uniformity of variability (Box M index = 158.127, F = 1.312, *p* = 0.1 > 0.05) was not statistically significant at the 95% confidence level, confirming homogeneity in sample variability across geographical origins. The first discriminant function accounted for 52.8% of the total variance, the second for 39.5%, and the third for 7.7%, collectively explaining 100% of the variance—a statistically optimal result. As depicted in Figure 1a, the regions analyzed were well differentiated. The overall correct classification rate was 92.9% for the original dataset and 82.1% for the cross-validation method, with the latter being considered particularly satisfactory.

### 3.2. Differentiation of Yellow Prickly Based on Elemental Composition

Similarly, the analysis demonstrated a significant multivariable effect of mineral composition on the geographical origin of yellow prickly pear, as indicated by Pillai’s Trace value of 2.603 (F = 2.764, *p* = 0.004 < 0.05) and Wilk’s Lambda value of 0.001 (F = 3.281, *p* = 0.003 < 0.05). Ten elements were found to be significant (*p* < 0.05) for distinguishing prickly pears from different regions and were subsequently analyzed using Linear Discriminant Analysis (LDA) (Table 2). The results revealed three statistically significant discriminant functions (Wilk’s Lambda = 0.029, X^2^ = 71.061, df = 30, *p* = 0.000 < 0.05 for the first; Wilk’s Lambda = 0.193, X^2^ = 32.884, df = 18, *p* = 0.017 < 0.05 for the second; and Wilk’s Lambda = 0.528, X^2^ = 12.779, df = 8, *p* = 0.012 < 0.05 for the third). The test for uniformity of variability (Box M index = 463.814, F = 146.468, *p* = 0.000 > 0.05) was not statistically significant at the 95% confidence level, confirming consistent sample variability across geographical origins. The first discriminant function accounted for 68.6% of the total variance, the second for 20.7%, and the third for 10.7%, collectively explaining 100% of the variance—an optimal result. As illustrated in Figure 1b, all regions were well differentiated. The overall correct classification rate was 92.9% for the original dataset and 75.0% for the cross-validation method, with the latter considered satisfactory.

Karabagias et al. (2019) [25] applied elemental analysis in combination with chemometrics to classify Greek yellow prickly pears from different regions in Greece, achieving a classification rate of 85.7%. Similarly, Albergamo et al. (2018) [40] reported a 100% classification rate for white Sicilian prickly pears based on their geographical origin. Compared to these studies, the classification rate obtained in the present study was lower.

### 3.3. Differentiation of Yellow Prickly Based on Antioxidants and Vitamins

The analysis demonstrated a significant multivariable effect of vitamins and antioxidants on the geographical origin of yellow prickly pear, as indicated by Pillai’s Trace value of 2.821 (F = 4.505, *p* = 0.000 < 0.05) and Wilk’s Lambda value of 0.000 (F = 8.117, *p* = 0.000 < 0.05). Sixteen compounds were found to significantly (*p* < 0.05) differentiate prickly pears from different regions and were subsequently analyzed using Linear Discriminant Analysis (LDA) (Table 3). The results revealed three statistically significant discriminant functions (Wilk’s Lambda = 0.000, X^2^ = 145.128, df = 48, *p* = 0.000 < 0.05 for the first; Wilk’s Lambda = 0.017, X^2^ = 68.809, df = 30, *p* = 0.000 < 0.05 for the second; and Wilk’s Lambda = 0.187, X^2^ = 28.535, df = 14, *p* = 0.012 < 0.05 for the third). The test for uniformity of variability (Box M index = 573.048, F = 109.152, *p* = 0.056 > 0.05) was not statistically significant at the 95% confidence level, indicating consistent sample variability across geographical origins. The first discriminant function accounted for 86.2% of the total variance, the second for 9.5%, and the third for 4.3%, collectively explaining 100% of the variance—an optimal result. As illustrated in Figure 1c, all tested regions were well differentiated. The overall correct classification rate was 100% for the original dataset and 96.4% for the cross-validation method, with the latter considered excellent.

El Guazzane et al. (2021) [41] determined the antioxidant activity of flowers, seeds, and cladodes of Moroccan prickly pears from four different regions. The classification rate based on this parameter was 96.3%, which is identical to the classification rate achieved in the present study.

LDA is a supervised technique, well-suited for classification tasks where group membership is known as an a priori, as is the case in this study. In contrast, PCA is an unsupervised method typically used when no prior information about group structure is available, which is not the case in the presented study. Moreover, LDA aims to maximize separation between predefined classes, aligning with our objective of origin-based differentiation, whereas PCA maximizes total variance, which may capture unrelated dimensions and potentially discard class-distinguishing features. Lastly, LDA provides interpretable linear combinations of variables that best separate groups, while MANOVA ensures that only statistically significant variables are included prior to classification.

## 4. Materials and Methods

### 4.1. Sample Collection

**Figure 2:**
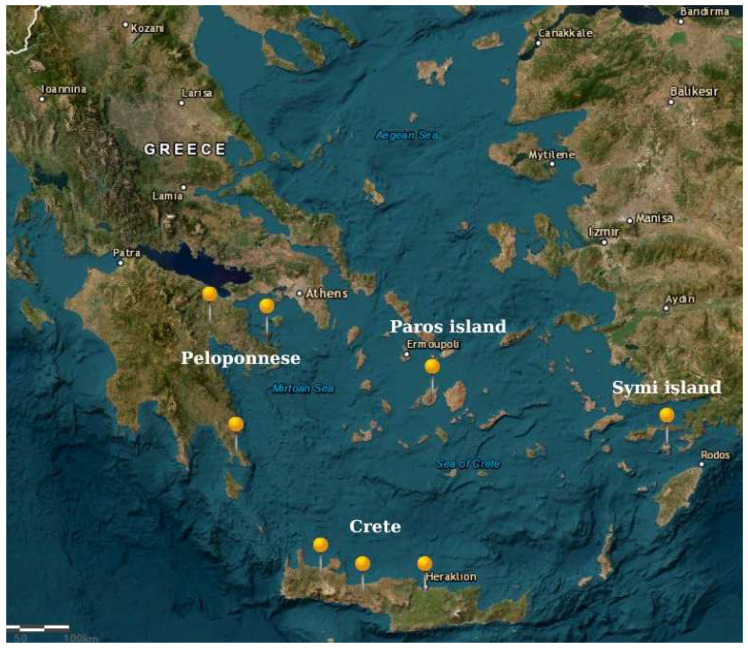
Map of Southern Greece with pins indicating the sample collection regions.

Fifty-six samples of yellow prickly pears from different geographical regions in Southern Greece were collected during the harvest periods of 2019 and 2020: Peloponnese (18), Crete (14), Symi Island (12), and Paros Island (12). The samples were collected by the authors from selected cooperatives or individual producers. Additionally, all samples were collected during the same maturity stage. After skin removal, prickly pear pulp samples were homogenized using a household mixer, transferred into sterilized 50 mL high-density polyethylene bottles, and stored at –80 °C until analysis. Further details on the sample origins and a map of the study areas are provided in Figure 2 and in Supplementary Material (Table S6).

### 4.2. Chemicals and Reagents

Anhydrous copper (II) sulfate, potassium sulfate, boric acid, sodium hydroxide, petroleum ether (40–60 °C, pro analysis), potassium hydroxide, and hydrochloric acid solution (both Titrisol, 1 mol/L), as well as phenolphthalein and methyl red indicators, were purchased from Merck (Darmstadt, Germany). The solvents acetonitrile (MeCN), methanol (MeOH), and water (H_2_O) (LC-MS grade) were also obtained from Merck (Darmstadt, Germany), while ammonium hydroxide solution (25% NH_4_OH) was sourced from Sigma-Aldrich (St. Louis, MO, USA). Hydrogen peroxide (H_2_O_2_, 30% for analysis) was acquired from Supelco (Darmstadt, Germany) and nitric acid (HNO_3_, 67–69% super pure for trace analysis) was from Carlo Erba (Sabadell, Spain). Acetic acid (CH_3_COOH, glacial, 100% anhydrous), 2,2-diphenyl-1-picrylhydrazyl (DPPH) free radical (PubChem CID: 74358), iron (II) sulfate heptahydrate (FeSO_4_·7H_2_O, PubChem CID: 62662), and sodium carbonate anhydrous (Na₂CO₃, PubChem CID: 10340) were obtained from Merck (Germany). Aluminum chloride hexahydrate (99%, AlCl_3_·6H₂O, PubChem CID: 24564), Folin and Ciocalteu’s phenol reagent (2 M, PubChem CID: 516996), and 2,4,4-tris(2-pyridyl)-5-triazine (≥98%, TPTZ, PubChem CID: 77258) were sourced from Sigma (USA). ±-6-Hydroxy-2,5,7,8-tetramethylchromane-2-carboxylic acid (Trolox, PubChem CID: 40634) was obtained from Aldrich (USA). Isorhamnetin (PubChem CID: 5281654), β-kaempferol (PubChem CID: 5280863, purity > 99%), and taurine (PubChem CID: 1123, purity >98%) were purchased from APExBIO (Houston, TX, USA), while luteolin (PubChem CID: 5281672), catechin (PubChem CID: 9064), quercetin (PubChem CID: 5280343, purity > 99%), rutin trihydrate (PubChem CID: 16218542), and gallic acid (PubChem CID: 370, purity > 94%) were obtained from Sigma (USA). Certified elemental standard solutions (1000 mg/L) for potassium (K), sodium (Na), calcium (Ca), magnesium (Mg), phosphorus (P), copper (Cu), and zinc (Zn) in 5% HNO_3_, as well as (100 mg/L) solutions for lithium (Li), beryllium (Be), strontium (Sr), titanium (Ti), vanadium (V), manganese (Mn), molybdenum (Mo), iron (Fe), cobalt (Co), nickel (Ni), platinum (Pt), aluminum (Al), silica (Si), tin (Sn), bismuth (Bi), selenium (Se), tellurium (Te), arsenic (As), lead (Pb), cadmium (Cd), barium (Ba), chromium (Cr), and boron (B) in 5% HNO₃, were purchased from CPAChem (Bulgaria). Certified reference material (grapefruit purée, FCCM23-FRU51RM) containing Cu = 612 μg/kg and Sn = 102 mg/kg was obtained from Fera Science Ltd. (York, UK).

### 4.3. Determination of Conventional Physicochemical Parameter Values

The determination of physicochemical parameters, including pH, moisture content, electrical conductivity (EC), titratable acidity (TA), ash content, crude protein, and fat, was performed according to official methods of analysis [42]. Total carbohydrate content was calculated by subtracting the sum of moisture content, ash content, crude protein, and fat from 100 [42]. The results are reported as the mean of three determinations (n = 3).

### 4.4. Determination of Color Parameters (L, α, b)

The surface color of yellow prickly pear pulp was measured using a HunterLab D25 L optical sensor (Hunter Associates, Reston, VA, USA). The colorimeter was calibrated with white and black standard plates. A 30 mL sample of yellow prickly pear pulp was placed into a cylindrical optical cell (base diameter: 11.3 cm, height: 2 cm). Reflectance values were obtained using a 45 mm viewing aperture. The results are reported as the mean of five determinations (n = 5).

### 4.5. Determination of Sugars Using UPLC-MS/MS

Chromatographic separation of sugars was performed using an ACQUITY I-Class LC System equipped with a triple quadrupole mass spectrometric detector (Xevo TQ-XS, Waters, Milford, USA), operating in gradient elution mode and coupled to a thermostatic autosampler (ACQUITY I-Class, Waters, USA). All operations were controlled by MassLynx Software (V4.2), and data acquisition was processed using TargetLynx (V4.1). Separation was achieved using an ACQUITY UPLC BEH Amide column (2.1 mm × 100 mm, 1.7 μm particle size, Waters, USA). The mobile phase consisted of 0.10% NH_4_OH in 30/70 MeCN/H_2_O (Phase A) and 0.10% NH_4_OH in 80/20 MeCN/H_2_O (Phase B). The optimized flow rate was set at 0.13 mL/min, with an injection volume of 5 μL. A linear gradient elution was applied, increasing the organic Phase B from 10% to 70% over 10 min. The B phase was then rapidly reduced to 10% within 0.01 min and maintained at this level from 10.01 to 12.00 min to equilibrate the column. The column temperature, initially varied between 35 °C and 50 °C for optimization, was set at 35 °C for final analysis. For MS/MS analysis, a triple quadrupole mass spectrometer equipped with an electrospray ionization (ESI) source was used. The instrument operated in positive ESI mode, switching to multiple reaction monitoring (MRM). Two prevalent transitions for each analyte (quantifier and qualifier ions) were selected for confirmation. The ion source parameters were set as follows: capillary voltage, 3.0 kV; cone voltage, 10.0 V; source temperature, 150 °C; desolvation gas temperature, 500 °C; desolvation gas flow, 600 L/h; and cone gas flow, 150 L/h (both using nitrogen gas).

### 4.6. Determination of Individual Vitamins Using UPLC-MS/MS

Chromatographic separation of vitamins was performed using the same instrumentation mentioned in Section 4.5. Separation was achieved using an ACQUITY UPLC HSS T3 column (2.1 × 100 mm, 1.8 μm particle size, Waters, USA). The mobile phase consisted of 10 mM ammonium formate with 0.1% formic acid in water (Phase A) and 10 mM ammonium formate with 0.1% formic acid in methanol (Phase B). The optimized flow rate was set at 0.45 mL/min, with an injection volume of 10 μL. A linear gradient elution was applied, where the organic phase B was increased from 1% to 5% within 0.1 min, further increased to 20% over the first 2.0 min, and then raised to 98% from 5.10 to 7.10 min. The 98% B phase was maintained from 7.10 to 9.0 min before being reduced back to 1% from 9.0 to 9.1 min to re-equilibrate the column. To optimize retention and selectivity, the column temperature was initially varied between 35 °C and 50 °C before being set at 40 °C for final analysis. For MS/MS analysis, a triple quadrupole mass spectrometer equipped with an electrospray ionization (ESI) source was used. The instrument operated in positive ESI mode, switching to multiple reaction monitoring (MRM). Two prevalent transitions for each analyte (quantifier and qualifier ions) were selected to confirm analyte identification. The ion source parameters were set as follows: capillary voltage, 3.0 kV; cone voltage, 10.0 V; source temperature, 150 °C; desolvation gas temperature, 500 °C; desolvation gas flow, 600 L/h; and cone gas flow, 150 L/h (both using nitrogen gas).

### 4.7. Determination of Individual Antioxidants Using UPLC-MS/MS

The determination of individual antioxidants was performed following the method described by Louppis et al. (2021) [43] without any modifications.

### 4.8. Determination of Total Phenolic Content (TPC), Total Flavonoid Content (TFC), and Antioxidant Activity

For the extraction of antioxidants, a 0.5 g portion of raw prickly pear was weighed into a 10 mL centrifuge tube. The samples were extracted with 5 mL of 80% aqueous methanol (MeOH), vortexed for 2 min, and sonicated for 40 min under controlled temperature (<15 °C). After centrifugation for 5 min at 10,000 rpm (10 °C), a specific volume of extract (E) was taken for each determination. The samples were analyzed using a UV V-730 spectrophotometer (Jasco, Tokyo, Japan). Total phenolic content (TPC) was determined using Folin and Ciocalteu’s reagent, following the method of Singleton and Rossi (1965) [44]. A reaction mixture was prepared by combining 200 μL of extract (E), 200 μL of Folin and Ciocalteu’s reagent, 1 mL of 8% (w/v) sodium carbonate solution, and 1.6 mL of distilled water. The mixture was vortexed for 1 min and allowed to react for 30 min. The absorbance was measured at 765 nm, and TPC was expressed as mg of gallic acid equivalent per kg of sample. Total flavonoid content (TFC) was determined according to Matić et al. (2017) [45]. Briefly, 800 μL of distilled water, 200 μL of extract (E), and 60 μL of 5% NaNO_2_ were added to a glass cuvette. After 5 min, 60 μL of 10% AlCl_3_ was added, followed by 400 μL of 1 mol/L NaOH and 480 μL of distilled water after 6 min. The absorbance was measured at 510 nm, and TFC was expressed as mg of quercetin equivalent per kg of sample. Total antioxidant activity (TA) was estimated using two different assays, the DPPH radical scavenging assay and the FRAP (Ferric Reducing Antioxidant Power) assay, according to Ozgen et al. (2006) [46]. For the DPPH assay, 100 μL of extract (E) was mixed with 2.9 mL of a methanolic DPPH solution and vortexed for 15 s. The mixture was incubated in the dark for 30 min, and the absorbance was measured at 515 nm. The results were expressed as mg of Trolox equivalent per kg of sample. For the FRAP assay, 400 μL of extract (E) was mixed with 3.6 mL of the FRAP reagent (TPTZ:FeCl_3_:CH_3_COOH, 1:1:10) and vortexed for 1 min. The mixture was incubated at 37 °C for 10 min. After centrifugation (5 min, 10,000 rpm), the sample was filtered through a PTFE filter (13 mm, 0.2 μm), and absorbance was measured at 593 nm. The results were expressed as mg of FeSO_4_ per kg of sample.

### 4.9. Determination of Minerals Using ICP-MS

For microwave digestion, a sample portion of 0.5000 ± 0.0010 g of raw prickly pear was weighed into a Teflon vessel. Aliquots of 1 mL of 30% hydrogen peroxide and 9 mL of 67–69% super pure nitric acid were added. The vessel was tightly sealed and placed into the ETHOS EASY microwave system (Milestone, Banemarksvej, Denmark). Digestion was initiated at 25 °C, with the temperature linearly increased to 210 °C at 1800 W. Samples were maintained at this temperature for 15 min. After cooling to room temperature, the digested sample was transferred into a 20 mL volumetric flask and diluted with distilled water. Elemental analysis was performed using an ICP-MS NexIon 1000 (Perkin Elmer, UK) under the following operating conditions: nebulizer gas flow, 1.02 L/min; auxiliary gas flow, 1.2 L/min; plasma gas flow, 15 L/min; analog stage voltage, −1637 V; sweep, 20; and replicates, 3.

### 4.10. Statistical Analysis

The SPSS v23.0 Statistics software (IBM Corp., Armonk, NY, USA) was used to perform Multivariate Analysis of Variance (MANOVA) and Linear Discriminant Analysis (LDA) following the approach of Louppis et al., (2023a) [26]. MANOVA was conducted to compare means and identify significant parameters (*p* < 0.05) for differentiating yellow prickly pear samples. To achieve differentiation based on geographical origin (independent variable), the data were analyzed to extract potentially important conventional physicochemical markers, including sugars, color, elements, antioxidants, and vitamins (dependent variables). The selected dependent variables were then utilized in LDA to assess the feasibility of classifying prickly pear samples according to their geographical origin. Both original and leave-one-out cross-validation methods were applied to evaluate the classification prediction accuracy.

## 5. Conclusions

This study provides an innovative approach to differentiating Southern Greek yellow prickly pear samples based on their geographical origin through an analysis of physicochemical parameters, selected vitamins, antioxidants, and minerals combined with advanced chemometrics. The classification success rates, 82.1% for physicochemical parameters, 75.0% for minerals, and 96.4% for vitamins and antioxidants, demonstrate the reliability and precision of such a differentiation. To the best of our knowledge, this is the first study to employ a multi-dimensional analytical strategy using both classical and advanced techniques for the comprehensive profiling of Southern Greek yellow prickly pears. Additionally, this is the first study to quantify and utilize a total of 34 chemical compounds for regional differentiation, identifying eight physicochemical parameters, ten minerals, and sixteen bioactive compounds as key markers. These findings highlight the potential of combining targeted analytical methodologies with chemometrics as a robust tool for food authentication, traceability, and quality control, offering new perspectives in the geographical characterization of Greek prickly pears.

## Figures and Tables

**Table 1:** Physicochemical parameters (average values ± standard deviation) in Greek yellow prickly pears (Crete, Symi, Paros, and Peloponnese).

Compound	Crete	Symi	Paros	Peloponnese
pH *	6.27 ± 0.11 ^ab^	6.19 ± 0.14 ^ab^	6.13 ± 0.19 ^a^	6.35 ± 0.14 ^b^
Electrical Conductivity * (μS/cm)	151 ± 26 ^a^	187 ± 28 ^ab^	202 ± 21 ^b^	173 ± 30 ^ab^
Titratable acidity (% as citric acid)	0.070 ± 0.0000	0.079 ± 0.029	0.064 ± 0.011	0.066 ± 0.011
Crude protein (g/100 d.w.b.)	0.91 ± 0.23	1.08 ± 0.31	0.79 ± 0.24	1.15 ± 0.42
Moisture content * (g/100 g)	78 ± 4 ^a^	84 ± 2 ^b^	83 ± 3 ^b^	84 ± 3 ^b^
Ash (acid insoluble, g/100 g)	0.55 ± 0.10	0.55 ± 0.04	0.62 ± 0.11	0.56 ± 0.10
Crude fat (g/100 d.w.b.)	2.27 ± 0.87	2.43 ± 0.85	2.40 ± 0.00	2.51 ± 0.83
Carbohydrates * (g/100 g)	18 ± 4 ^b^	11 ± 1 ^a^	14 ± 3 ^a^	11 ± 3 ^a^
Fructose * (g/100 g)	2.86 ± 1.04 ^b^	1.25 ± 0.61 ^a^	3.69 ± 1.17 ^b^	2.42 ± 0.52 ^ab^
Glucose * (g/100 g)	3.73 ± 1.44 ^b^	0.82 ± 0.06 ^a^	4.36 ± 1.37 ^b^	1.53 ± 0.88 ^a^
L	65.68 ± 1.21	65.40 ± 3.46	61.34 ± 2.59	62.66 ± 4.20
a *	−1.54 ± 1.27 ^ab^	−3.21 ± 1.42 ^a^	0.91 ± 3.32 ^b^	−1.79 ± 1.86 ^ab^
b *	0.91 ± 0.30 ^ab^	1.04 ± 0.56 ^b^	0.45 ± 0.36 ^a^	0.78 ± 0.40 ^ab^

^a,b^ mean values for geographical differentiation with different superscripts in the same row are significantly different * *p*-values are the result of the application of MANOVA (*p* < 0.05) to data for geographical differentiation.

**Table 2:** Mineral content (average values ± standard deviation) in Greek yellow prickly pears (Crete, Symi, Paros and Peloponnese).

Mineral	Crete	Symi	Paros	Peloponnese
K	1794 ± 311	2237 ± 151	2216 ± 372	1893 ± 408
Ca	349 ± 109	384 ± 151	566 ± 269	383 ± 134
P	249 ± 131	360 ± 105	184 ± 70	322 ± 130
Mg	221 ± 114	231 ± 77	272 ± 84	270 ± 93
B *	4.65 ± 1.41 ^b^	4.81 ± 0.53 ^b^	3.54 ± 0.48 ^ab^	3.31 ± 0.70 ^a^
Si *	1.61 ± 0.36 ^a^	2.66 ± 0.38 ^ab^	2.84 ± 0.95 ^ab^	3.24 ± 1.13 ^b^
Zn	1.99 ± 1.37	3.44 ± 1.20	1.79 ± 0.71	2.82 ± 1.16
Mn *	5.17 ± 0.56 ^ab^	2.67 ± 1.25 ^a^	1.27 ± 0.62 ^a^	10.00 ± 6.04 ^b^
Na *	4.09 ± 0.83 ^ab^	3.45 ± 0.62 ^a^	6.09 ± 2.57 ^b^	3.32 ± 0.84 ^a^
Fe	1.99 ± 2.35	2.04 ± 0.60	1.38 ± 0.46	2.22 ± 0.77
Sr *	0.36 ± 0.21 ^a^	0.281 ± 0.08 ^a^	0.26 ± 0.13 ^a^	0.51 ± 0.22 ^a^
Al *	0.52 ± 0.26 ^a^	0.23 ± 0.07 ^a^	0.91 ± 0.43 ^b^	0.23 ± 0.09 ^a^
Cu *	0.37 ± 0.17 ^a^	0.58 ± 0.14 ^a^	0.34 ± 0.13 ^a^	0.52 ± 0.19 ^a^
Ni *	0.48 ± 0.17 ^ab^	0.61 ± 0.22 ^b^	0.20 ± 0.16 ^a^	0.68 ± 0.25 ^b^
Ba *	0.12 ± 0.06 ^a^	0.27 ± 0.14 ^ab^	0.14 ± 0.12 ^a^	0.46 ± 0.34 ^b^
Sn *	0.07 ± 0.01 ^ab^	0.03 ± 0.00 ^a^	0.14 ± 0.09 ^b^	0.03 ± 0.00 ^a^
Ti	0.030 ± 0.006	0.052 ± 0.036	0.015 ± 0.002	0.023 ± 0.003
Mo	0.022 ± 0.003	0.023 ± 0.014	0.010 ± 0.005	0.014 ± 0.008
Co	0.004 ± 0.002	0.003 ± 0.001	0.003 ± 0.001	0.002 ± 0.000

^a,b^ mean values for geographical differentiation with different superscripts in the same row are significantly different * *p*-values are the result of the application of MANOVA (*p* < 0.05) to data for geographical differentiation.

**Table 3:** Vitamin and antioxidant content (average values ± standard deviation) in Greek yellow prickly pears (Crete, Symi, Paros, and Peloponnese).

Compound	Crete	Symi	Paros	Peloponnese
DPPH * (mg/kg Trolox)	9 ± 2 ^a^	7 ± 4 ^a^	16 ± 2 ^b^	4 ± 1 ^a^
FRAP * (mg/kg FeSO_4_)	41 ± 5 ^a^	48 ± 11 ^a^	62 ± 8 ^b^	50 ± 5 ^a^
Total flavonoids * (mg/kg quercetin)	4.07 ± 0.32 ^a^	4.67 ± 0.61 ^ab^	6.20 ± 1.97 ^b^	4.72 ± 0.96 ^ab^
Total phenolics * (mg/kg gallic acid)	114 ± 19 ^c^	66 ± 10 ^a^	90 ± 12 ^b^	70 ± 7 ^a^
Nicotinic Acid * (μg/kg)	70 ± 34 ^ab^	105 ± 34 ^b^	27 ± 18 ^a^	177 ± 62 ^c^
Nicotinamide (μg/kg)	16 ± 7	27 ± 18	22 ± 17	22 ± 14
Pyridoxal (μg/kg)	14 ± 5	18 ± 9	10 ± 6	12 ± 4
Pyridoxine * (μg/kg)	2.25 ± 0.72 ^a^	3.47 ± 1.30 ^a^	2.12 ± 1.31 ^a^	6.38 ± 1.48 ^b^
Vitamin B5 * (μg/kg)	754 ± 387 ^a^	927 ± 177 ^ab^	630 ± 341 ^a^	1364 ± 228 ^b^
Vitamin B7 (μg/kg)	23 ± 11 ^a^	25 ± 5 ^a^	20 ± 11 ^a^	23 ± 6 ^a^
Vitamin B1 * (μg/kg)	10.50 ± 5.60 ^ab^	8.59 ± 2.57 ^ab^	15.90 ± 10.04 ^b^	6.40 ± 1.60 ^a^
Vitamin B2 * (μg/kg)	50 ± 20 ^a^	53 ± 20 ^a^	30 ± 17 ^a^	31 ± 9 ^a^
Vitamin C * (μg/kg)	69272 ± 22628 ^b^	27309 ± 17302 ^a^	69054 ± 43416 ^b^	8907 ± 6919 ^a^
Taurine * (μg/kg)	1.48 ± 0.54 ^ab^	1.12 ± 0.17 ^a^	2.12 ± 0.78 ^b^	2.43 ± 0.81 ^b^
Rutin * (μg/kg)	36 ± 16 ^b^	34 ± 14 ^b^	35 ± 15 ^b^	6 ± 1 ^a^
Quercetin * (μg/kg)	19 ± 9 ^b^	4 ± 3 ^a^	14 ± 5 ^ab^	24 ± 13 ^b^
Catechin * (μg/kg)	21 ± 12 ^b^	9 ± 6 ^b^	11 ± 3 ^ab^	3 ± 2 ^a^
Isorhamnetin * (μg/kg)	12.5 ± 8.6 ^ab^	0.7 ± 0.5 ^a^	7.0 ± 6.1 ^a^	21.0 ± 11.7 ^b^
Gallic acid (μg/kg)	0.96 ± 0.22	0.49 ± 0.48	0.59 ± 0.42	1.39 ± 1.01
Kaempferol * (μg/kg)	5.87 ± 3.20 ^bc^	0.45 ± 0.04 ^a^	2.99 ± 2.45 ^ab^	8.28 ± 2.64 ^c^

^a,b,c^ mean values for geographical differentiation with different superscripts in the same row are significantly different * *p*-values are the result of the application of MANOVA (*p* < 0.05) to data for geographical differentiation.

## Data Availability

The data will be available upon request.

## Supplementary Materials

The following supporting information can be downloaded at: https://www.mdpi.com/article/10.3390/molecules30112448/s1. Table S1: Information on origin of prickly pear samples; Table S2: Calibration curves, linear regressions, limit of detections (LODs), recoveries for quality control samples (QC) and CRM sample for minerals; Table S3: Calibration curves, linear regressions, matrix effect and carryover for all compounds; Table S4: Limits of detection, limits of quantification, retention times, and recoveries at three different concentrations for all analytes in prickly pear (n = 6 samples); Table S5: Method performance data at three different concentrations for all analytes; Table S6: Information on origin of prickly pear samples.
