# Supplementary material for: Chemical Markers for Differentiating Yellow Prickly Pear (Opuntia ficus-indica) from Southern Greece: Insights from Physicochemical Parameters, Elemental Composition, Antioxidants, and Vitamins
Source: Molecules. 2025 Jun 3;30(11):2448. doi: 10.3390/molecules30112448 (PMC12156975; doi:10.3390/molecules30112448)
Supplement: Supplementary file 1 [file molecules-30-02448-s001.zip › molecules-3683387-supplementary.pdf]

Supplementary material

Table S1. Calibration curve equations and R^2 for sugars

	Calibration curve	R^2
Glucose	Y=2773x-145	0.9995
Fructose	Y=2888x-369	0.9999

Table S2. Calibration curves, linear regressions, limit of detections (LODs), recoveries for quality control samples (QC) and CRM sample for minerals.

**Mineral**	**Calibration curve**	**Linear Regression (R^2)**	**LOD (mg kg^{-1})**	**Recovery of QC (%)**	**Recovery of CRM (%)**
K	Y=22868x	0.999948	4	98	-
Ca	Y=26257x	0.999957	4	92	-
P	Y=3257273x	0.999963	4	99	-
Mg	Y=17253x	0.999949	4	95	-
B	Y=7892x	0.998653	0.1	89	-
Si	Y=101x	0.991357	0.1	90	-
Zn	Y=476x	0.997623	0.1	89	-
Mn	Y=758x	0.999889	0.1	88	-
Na	Y=71x	0.990118	0.1	90	-
Fe	Y=3080x	0.999685	0.1	92	95
Sr	Y=3163x	0.999927	0.1	95	-
Al	Y=78071x	0.998833	0.1	99	-
Cu	Y=5390x	0.999532	0.1	90	92
Ni	Y=1958x	0.999983	0.1	97	-
Ba	Y=117484x	0.999333	0.1	98	-
Sn	Y=532x	0.999952	0.001	89	88
Ti	Y=171x	0.997782	0.001	88	-
Mo	Y=60408x	0.998268	0.001	85	-
Co	Y=1542x	0.999847	0.001	86	-

Table S3. Calibration curves, linear regressions, matrix effect and carryover for all compounds.

Analyte	Calibration curve	Linear Regression, (R^2)	Matrix Effect (mean ± SD, %)	Carryover (%)
Ascorbic acid	y = 777x - 2331	0.9999	64 ± 8	1.2

Catechin	y = 12782x - 2483	0.9971	68 ± 12	0.1
Gallic acid	y = 2827x - 728	0.9969	111 ± 14	1.4
Isorhamnetin	y = 21926x - 4416	0.9997	65 ± 8	2.7
Kaempferol	y = 6516x - 3486	0.9966	66 ± 9	0.8
Luteolin	y = 8228x - 2152	0.9987	79 ± 12	0.0
Myricetin	y = 1461x - 1725	0.9916	50 ± 8	0.0
Quercetin	y = 5365x - 1550	0.9930	77 ± 19	2.9
Rutin	y = 11546x - 2628	0.9996	47 ± 10	0.3
Taurine	y = 1157x - 1344	0.9987	52 ± 11	0.6

Table S4. Limits of detection, limits of quantification, retention times, and recoveries at three different concentrations for all analytes in prickly pear (n = 6 samples).

				Recovery (%)		
Analyte	LOD (µg /kg)	LOQ (µg /kg)	Retention time (min)	Conc. A	Conc. B	Conc. C
Ascorbic acid	15.0	45.4	0.850 ± 0.004	102.1	78.2	94.2
Catechin	0.21	0.62	3.762 ± 0.002	107.2	86.0	85.8
Gallic acid	0.55	1.66	2.569 ± 0.004	74.3	84.1	83.0
Isorhamnetin	0.17	0.50	5.280 ± 0.006	101.7	75.0	70.9
Kaempferol	0.18	0.54	5.226 ± 0.004	109.2	86.5	83.4
Luteolin	0.35	1.06	5.105 ± 0.034	97.3	62.2	68.0
Myricetin	0.80	2.41	4.714 ± 0.004	77.6	98.3	100.1

Quercetin	0.82	2.45	5.006 ± 0.000	112.4	103.6	86.3
Rutin	0.49	1.48	4.521 ± 0.003	87.4	104.0	94.8
Taurine	0.61	1.72	0.624 ± 0.003	100.5	101.4	88.7

Conc. A: 3 µg/kg for all antioxidants and 50 µg/kg for ascorbic acid, Conc. B: 30 for all antioxidants and 1000 µg/kg for ascorbic acid, Conc. C: 80 for all antioxidants and 2000 µg/kg for ascorbic acid.

Table S5. Method performance data at three different concentrations for all analytes.

	Conc. A				Conc. B				Conc. C			
Analyte	Mean concentration (µg/ kg)	SD	RSD (%)	MU (%)	Mean concentration (µg/ kg)	SD	RSD (%)	MU (%)	Mean concentration (µg/ kg)	SD	RSD (%)	MU (%)
Ascorbic acid	51.05	1.05	9.84	10.1	782	12.3	8.12	9.8	1884	97.5	7.02	8.6
Catechin	3.20	0.00	0.15	12.4	25.80	0.85	3.35	12.8	68.70	3.85	5.64	10.0
Gallic acid	2.25	0.20	8.94	15.0	25.25	0.55	2.15	13.9	64.0	4.35	6.52	9.8
Isorhamnetin	3.05	0.25	7.92	12.6	22.50	0.35	1.46	9.7	56.75	0.85	1.49	9.7
Kaempferol	3.30	0.20	6.12	19.0	25.95	2.25	8.58	14.1	66.75	5.20	7.76	11.9
Luteolin	2.90	0.35	11.40	14.9	18.65	0.65	3.50	11.2	54.35	3.15	5.78	10.0
Myricetin	2.35	0.25	10.63	15.2	29.50	0.70	2.36	13.2	80.05	5.05	6.34	9.8
Quercetin	3.35	0.20	6.04	12.7	31.05	2.70	8.67	17.0	69.00	3.40	4.93	11.9
Rutin	2.60	0.30	12.29	11.7	31.20	1.20	3.88	11.1	75.80	3.25	4.31	10.1
Taurine	3.01	0.28	10.55	13.2	30.42	0.99	1.59	14.0	70.96	4.89	4.22	10.4

Conc. A: 5, 50 µg/ kg for all antioxidants ascorbic acid, respectively, Conc. B: 30 and 1000 µg/ kg for all antioxidants ascorbic acid, respectively, Conc. C: 80 and 2000 µg/kg for all antioxidants ascorbic acid, respectively.

Table S6. Information on origin of prickly pear samples.

Country	Variety	Area
Greece	Yellow	Crete (Chania)
		Crete (Chania)
		Crete (Rethimno)
		Crete (Rethimno)
		Crete (Rethimno)
		Crete (Irakleio)
		Crete (Irakleio)
		Crete(Irakleio)
		Symi Island
		Symi Island
		Symi Island
		Symi Island
		Symi Island
		Symi Island
		Paros Island
		Paros Island
		Paros Island
		Paros Island
		Paros Island
		Paros Island
		Peloponnese (Argos)
		Peloponnese (Argos)
		Peloponnese (Argos)
		Peloponnese (Methana)
		Peloponnese (Methana)
		Peloponnese (Methana)
		Peloponnese (Neapolis)
		Peloponnese (Neapolis)
		Peloponnese (Neapolis)
